# Abstracts and Gleanings

**Published:** 1884-11-20

**Authors:** 


### ABSTRACTS AND GLEANINGS.

**Pneumonia, Bronchitis and Pleurisy Treated Antiseptically.**—Dr. Wm. F. Hamer reports these cases in the St. Louis Courier of Medicine for April, 1884:

CASE 1.

B. S., aged 23, was taken with a chill; soon followed fever, oppressed breathing, and all the symptoms of pneumonia. When I saw him his pulse was 115, temperature 103°. The bowels being constipated, I ordered as an enema a quart of warm water, with a teacup of sweet milk, and about a tablespoonful of common salt added to it—all of this to be thrown into the bowels at once. This relieved them in a few minutes. I made counter-irritation over the affected part with ground flaxseed, sprinkled with mustard and made into a poultice, and gave the following internally:

℞ Quiniæ sulph....................................grs. xx,
Pulv. iodoform (pulverized very fine).........grs. x,
Granulated acacia.............................grs. vj.
M. Triturate well in a mortar, and make capsules No. x.
Sig. One every three hours, alternately with:

℞ Sodæ hyposulphitis..........................ʒ jss,
Tinct. verat. viridis .........................gtt. xviij,
Syr. pruni virg...............................℥ iij.
M. Sig. Teaspoonful every three hours. Shake well.

At my next visit, which was ten hours from the first: Pulse 110, temperature 101¼°. Suffering very much relieved. In two days expectoration commenced, having its usual character, rusty sputa. The height of the attack was reached by the fifth day, and on the eleventh day he was out on the street, feeling, as he expressed it, "all right." This was all the treatment used in this case, and four others of about the same nature which it is not necessary to relate, only some whisky was used to make some panada and egg nog. These patients had less pain, less cough and fever, and all made good and speedy recoveries. At the same time I had other cases of pneumonia and treated them in the old way, as I call it, antiphlogistically, and giving cough sirups of different kinds, carbonate of ammonia, etc. They were more tedious, suffered more pain, and did not recover under fourteen and twenty days.

CASE 2.

Miss S., aged 18, had had a chill, and was suffering with sharp pain in the side, accelerated respiration, short cough and fever. Diagnosis, pleurisy. The bowels were opened with sulphate of magnesia. To the painful part an application was made of equal parts ef belladonna and aconite liniment, covered with a hot sandbag: internally,

℞ Sodæ hyposulphitis ........................ ʒ jss,
Tinct. verat. viridis ........................ gtt. xviij,
Syr. scillæ ................................ ℥ iij.
M. Sig. Teaspoonful every two or three hours. Shake the bottle well before pouring it out.

This was to be given alternately with the same capsules as in the pneumonia cases. I saw her in eight hours. Respiration was easier, pain less, and all the symptoms better. Instead of the sand-bag, a flax-seed poultice was used. This treatment was continued, and she made a speedy recovery. Two other cases were treated in the same way, with like results. In fact, they all recovered quicker than by any other treatment that is recommended.

CASE 3.

Mr. L., aged 29, called me to see him. I found him depressed, and having high fever, tightness across the upper and anterior part of the chest, and short, sharp, dry cough. The bowels being constipated, I ordered a dose of Rochelle salts, and covered the upper sternal region with a large linseed poultice, sprinkled with mustard. I also ordered inhalations of steam, combined with hops and a little carbolic acid. Internally, I gave the same capsules as before, and the following drops:

℞ Sodæ hyposulphitis ........................ ʒ jss,

Tinct. aconiti radicis ........................ gtt. xviij,

Syr. scillæ,

" " pruni virg., } aa .................. ℥ jss,

M. Sig. Teaspoonful every two or three hours.

This was continued twenty-four or thirty-six hours. The patient was seen but once, and made a speedy recovery.

Now, this is what I call the antiseptic treatment, and these are septic troubles.—*Med. and Surg. Reporter.*

**The Compass Plant.**—The following interesting account, by Sir J. D. Hooker, of the compass plant (*Silphium laciniatum*) of the Western prairies is taken from Curtis's Botanical Magazine:

"This noble plant was introduced (from America) into Europe in 1781 by M. Thouin, and flowered for the first time in the Botanic Garden of Upsala, in Sweden. It has been in cultivation in Europe ever since, though its name and fame as the compass plant of the prairies are of comparatively modern date, it having before that borne the popular names of turpentine plant and rosin-weed, except among the hunters and settlers in the Western States. With regard to the history of its reputed properties as an indicator of the meridian by the position of its leaves, I am fortunate in having recourse to my friend, Professor Asa Gray, now in England, who has most kindly furnished me with the following very interesting account of the matter:

"'The first announcement of the tendency of the leaves of the compass plant to direct their edges to the north and south was made by General (then Lieutenant) Alvord, of the U. S. Army, in the year 1842, and again in 1844, in communications to the American Association for the Advancement of Science. But the fact appears to have been long familiar to the hunters who traversed the prairies in which this plant abounds. The account was somewhat discredited at the time, by the observation that the plants cultivated at the Botanic Garden at Cambridge, U. S., did not distinctly exhibit this tendency. But repeated observation upon the prairies, with measurements by the compass of the directions assumed by hundreds of leaves, especially of the radical ones, have shown that, as to prevalent position, the popular belief has a certain foundation in fact.

**The Missing Link of the Cholera Germ Found at Last.**—In his communications, thus far, Koch has been unable to show that his comma bacilli did produce cholera. Nor has he been able to demonstrate the early life-history of the bacillus. In the British Medical Journal, September 6th, we have the completion of the life-history of the bacillus, given by Drs. Maurin and Lange. These observers have continued working at Marseilles and report that they have found a mucor, which they regard as the actual agent in propagating cholera. This mucor appears on the fourth or fifth day on the putrefying stools of cholera, and on these only. It has the form of a mycelium, the tapering ends of which are surmounted by cup-shaped sporangia, which burst on the slightest agitation, discharging vast numbers of spores. These spores require for their germination contact with some putrid organic matter, when they develop into mucor of another form, an anærobium which they believe to be the immediate cause of the phenomena of the disease, and which again, in its turn, sporifying, gives birth to the bacilli of Koch.

While the bacilli themselves are inocuous, when deposited on putrid matter in the air they develop the first mentioned mucor, and so renew the cycle.

The first mucor, unlike the bacillus, has the power in a high degree of resisting the action of so-called disinfectants. It is not killed by 10 per cent. solutions of nitric or hydrochloric acids. It vegetates freely in a solution of equal strength of carbolic acid. It can sustain any temperature up to three hundred and two degrees F., but above this it breaks up, as it also does in a 10 per cent. solution of tincture of iodine. A specimen mounted in oil of turpentine went through its whole development up to the discharge of its spores.

The verification of these claims is looked for with the greatest interest.—*Detroit Lancet.*

**Fever and Kairin; Plethysmographic Researches.**—Under the direction of Professors Maragliano and Mosso, Dr. Queirolo, of Genoa, has made some researches on the conditions of the peripheral blood-vessels in fever, and on the changes produced in them by the administration of kairin in healthy individuals and those suffering from pyrexial affections. The researches, which appear to have been carefully conducted, gave the following results:

1. Rise of temperature is accompanied by narrowing of the peripheral blood-vessels.

2. The contraction of the blood-vessels precedes the initial rise of temperature, and dilatation of them precedes the fall.

3. The narrowing of the blood-vessels is greater in proportion to the height and rapidity of rise of temperature.

4. The defervescence of the fever is accompanied by dilatation of the peripheral blood-vessels.

5. The dilatation of the blood-vessels slightly precedes the defervescence.

6. Any interruption to the dilatation of the blood-vessels corresponds to an interruption in the fall of temperature.

7. These facts are observable in fevers of various kinds.

With regard to kairin—

1. Kairin administered to healthy individuals causes slight dilatation of peripheral blood-vessels.

2. In individuals suffering from fever, kairin produces diminution of temperature, and at the same time dilatation of vessels.

3. The dilatation of blood-vessels slightly precedes the fall of temperature.

4. Subsequent rise of temperature after kairin has expended its force is accompanied by contraction of blood-vessels.

5. The rise of temperature is slightly preceded by dilatation (contraction?) of the blood-vessels.

If these observations prove to be correct, it will be difficult to overestimate their value, as they may possibly lead the way to more clear and correct views as to the origination of fever.—*The Med. Press.*

**Treatment of Asthma.**—Dr. Robert Saundby (Birmingham Medical Review), in the course of an interesting paper on the subject of asthma, says: Is there any drug that wards off the attacks? This is a very important question, and one which I have set myself to answer. Belladonna, arsenic, lobelia, and iodide of potassium, have each found their supporters. I have given a sufficient trial to all of these, and the only one in which I have any confidence is iodide of potassium. Its value in this disorder has been long known, but it is not so generally appreciated as it should be. This may partly be because the dose required is large, ten grains three times a day, or the same amount in two doses of fifteen grains each. Another drug, which certainly appears to be of use, is sulphur, the balsamum pectoris of the celebrated Hoffman. This may be given in doses of ten or twenty grains in sirup or honey, once or twice daily.—*Louisville Med. News.*

**Prevention of Ophthalmia Neonatorum.**—One of the greatest blessings which medical art of late has conferred on mankind is the preventive treatment of the blenorrhœa of the eyes of newborn children. In the October number of the American Journal of the American Sciences, Dr. Henry J. Garrigues calls attention to the value of Crede's method of treatment, which, in brief, consists in washing the outer surface of the eyelids with plain water, separating them slightly and letting a single drop of a two-per-cent solution of nitrate of silver fall from a glass rod on the cornea. No after-treatment is used.

In 1882 Garrigues introduced Crede's treatment into his service at the New York maternity hospital, since which time three hundred and fifty-one children have been thus treated and not a single one was affected. He makes this application immediately after the cord has been severed, which is not done until the pulsation in it has ceased. During the subsequent ablution great care is taken that no foreign substance enters the eye.

The results in lying-in hospitals by this method are so striking that there cannot be any doubt about the advisability, nay, the duty of adopting it in all such institutions.—*Med. Age.*

**The Paste Treatment of Inflammatory Skin Diseases, Especially Eczema.**—Dr. P. G. Unna (Monatshefte fur Practische Dermatologie; Edinburgh Med. Jour.), finding the greasiness and expense of ointmentsto bestrong objections to their use, found that he could use a kaoline paste to advantage, and soon found that lead, starch, dextrin and gum pastes might replace the kaoline paste. A good paste should be quickly and easily spread as a thin layer on the skin, and should spontaneously form, in a short time, a dry, firmly adherent coating.

### KAOLINE PASTES.

Pure kaoline, with vaseline or glycerine in equal parts, with oils such as olive, almond, linseed, in the proportion of 2 to 1, will produce a good paste. With more linseed oil a liniment is produced. This, spread on extensive surfaces, leaves a quickly drying residuum. When other ingredients, such as acetate of lead or oxide of zinc, are used, the kaoline and oil or glycerine are first to be mixed, and then the lead or zinc added, as the kaoline and the mineral are otherwise apt to form an insoluble cement. A suitable paste for eczema is this:

| | | |
|---|---|---|
| ℞ | Kaolini puri,<br>Ol. lini (v. glycerini) | aa........................30-0 |
| | Zinci oxidi,<br>Liq. plumbi subacet, | aa........................20-0 |

Instead of white kaoline, the yellow or red is occasionally preferable for pastes for the face. The kaoline pastes are not merely suitable for the treatment of all kinds of eczemas, erythemata, and intertrigines, but are also suited as vehicles for strongly oxidizing, reducing or caustic remedies.

### LEAD PASTES.

Boil a quantity of litharge with double the quantity of vinegar till the vinegar has evaporated and the litharge has been transformed into a moderately damp paste. Should the paste become dry in time, it can be heated up with a fresh quantity of vinegar. This paste possesses considerable exsiccative power.

| | |
|---|---|
| ℞ Lithargyri subt. pulv | 50-0 |
| Aceti | 80-0 |

Boil to the consistency of paste and add

| | |
|---|---|
| Ol. lini (v. glycerini, v. ol. olivæ) | 10-0 |

### STARCH PASTES.

Here the property of drying must be imparted by the addition of some tempering material, as oxide of zinc, sulphur, etc. The following forms a good starch paste for eczema:

| | |
|---|---|
| ℞ Zinci oxidi | 50-0 |
| Acid salicylici | 2-0 |
| Amyli oryzæ | 15-0 |
| Glycerini | 15-0 |
| Aq. distill | 75-0 |

Mixed directly and all heated at once to 140-0.

Starch paste for acne:

| | |
|---|---|
| ℞ Sulphuris precip | 40 0 |
| Calcis carbonat | 2-0 |
| Zinci oxidi | 20-0 |
| Amyli oryzæ | 15-0 |
| Glycerini | 20-0 |
| Aq. distill | 75-1 |
| M. Boil to | 120-0 |

—*Journal Amer. Med. Association.*

**The Treatment of Pernicious Intermittent Fever.**—Dr. W. H. Reed reports an interesting case, which yielded to treatment, in the College and Clinical Record.

His treatment was as follows: Immediately a hypodermic injection of one-sixth grain of morphiæ sulph.; hot sand-bag to lumbar region; one pil. podophyllin. comp., and two grains of quiniæ sulph., in pills, every two hours, until twenty grains were taken. He was sent for hurriedly, with the statement that his patient was dying. On his arrival by her bedside, he found her in a sinking condition; in the meantime she had had several convulsions; was now at times in a low delirium, muttering inaudible and unconnected sentences, and picking at the bed-clothes. Her countenance was relaxed, tired, worn and haggard; skin somewhat cooler, and pulse almost imperceptible; heart sounds feeble; respiration slow and gasping. The pain had left the back, from the application of the sand-bag. He immediately stimulated her freely with whisky, which at first produced retching, but not vomiting; then he was informed that, on two occasions previously, she had vomited blood. He ordered dry heat over her stomach, which successfully allayed vomiting and retching, thereby enabling her to retain all medicine administered. A strong milk punch was given at intervals during the night, and the quinine pills continued as before ordered. Next day he found his patient much improved. She was lying quietly in bed, her mind clear and buoyant, but she was much exhausted and debilitated. The delirium passed off about midnight, and her bowels moved freely toward morning. Her skin was almost normal in temperature, and but slightly jaundiced; heart sounds regular, but slightly feeble; respiration normal; no thirst; tongue coated with a rather heavy fur. She ate some prepared gluten and milk for breakfast. He prescribed—

℞ Hydrargyri chloridi mitis......................grs. ij,
Pulv. ipecac..............................grs. ij,
Pulv. ingluvin...............................℈ j,
M. Fiant chartæ viij. Sig. One to be taken every two hours.

He also gave her ten-drop doses of Fowler's solution three times a day. When he saw her again two days later, his patient was out of bed, with skin comparatively clear; appetite returned, and she was commencing to feel strong again. At the septenary periods he prescribed twenty-four grains of cinchoniæ sulphas. One week later he diminished the Fowler's solution to five drops three times a day. Two weeks later he saw his patient. She was feeling exceedingly well, with no symptoms of a recurrence of the chill.—*Med. and Surg. Reporter.—Med. Digest.*

**Tetanus Treated with Quinine and Chloral.**—Dr. James H. Tebbetts, of Chicago, Ill., relates the history of a child, two months old, who had pushed a carpet tack, with a leather head, up its nose. Several days later Dr. Tebbetts was called in and found the child in tetanic convulsions. It had had trismus for two days already. The tack was found wedged against the inferior turbinated bone and was extracted, when about a drachm of sanious pus and broken down tissue was discharged. The convulsions continued, the temperature was 106° F., and pulse 170. Eight grains of chloral hydrate and five grains of quinine were given per rectum, when the general convulsions ceased, but not the trismus. Next morning the temperature was 104°. Ordered three grains of chloral hydrate every three hours for twenty-four hours, and five grains of quinine every six hours per rectum. This last medicine was continued for three days. At the end of that time the temperature was normal and there was only a slight trismus. Dr. Tebbetts adds: "It would seem that in quinine we are to look for great assistance in these cases, and that, when given in full doses, even in children, the convulsions lessen in severity and may cease altogether. Toy-pistol tetanus, in the practice of Dr. Hosmer Johnson, has been cured by large doses of quinine, and Dr. Jones, of New Orleans, also reports recoveries of cases of tetanus after large doses of quinine. In conjunction with chloral hydrate during and after the severer paroxysms, quinine seemed to hold the disease in check until the system had recovered its tone. Cinchonism did not appear."—*N. Y. Med. Record.*

**Treatment of Fracture of the Clavicle**—Dr. I. W. Chisholm (Col. Med. Jour.) says: "Being called to attend Mrs. Rev. B.

(who had fallen down stairs), I found, upon examination, a fracture of the right clavicle—towards the acromial extremity—and knowing from past personal experience (and also from the experience of other members of the profession) the difficulty of adjusting a bandage so as to hold the fractured ends in juxtaposition, the thought occurred to me of trying the following plan: Placing a small ball of candle-wick and pad in the axilla in the usual manner, then taking an ordinary muslin bandage, about 3½ or 4 feet long, and passing this around and underneath the axilla and drawing it firmly upward against the axillary pad; then crossing the two ends of the bandage on top of the injured shoulder (having placed a compress at the seat of fracture), and then taking one end of the bandage and passing it across the back and around and underneath the axilla of the uninjured side and across the chest, and drawing it tightly and uniting the two ends. Then placing the arm in a sling supported by a band passing from the sling upward and over the seat of fracture and across the back, around and under the axilla (of the uninjured side), and across in front of the chest to the sling, and securing it there. Then taking a small, firm band of muslin, and securing it to the sling at the elbow and fastening the other end to the band across the back.

The advantages I claim for this appliance are its comparative simplicity, the material being always at hand for construction, the fractured ends being doubly secured by pressure being made on them by both the bandage and the band of the sling, and also the shoulder being doubly supported by the axillary pad and the band-strap.

In the case in which I tried this appliance the bone united without any appreciable deformity whatever, it being impossible to discern at what point the fracture occurred.

I am not able to say whether this appliance will prove equally successful in every case in which it may be tried, but this I think I can confidently say; it has all the advantages of at least many other appliances, without some of the objectionable features; among which I might mention: "costliness," difficulty of mechanical construction, requiring the surgeon to keep them constantly on hand, unless within reach of the "instrument maker;" and another, the uninjured side is not required to pay penance by being bound, and its freedom of motion to a great extent restrained, for the misfortune of its fellow, which advantages are worthy of at least some consideration on the part of the members of the profession.—*Amer. Med. Digest.*

**A New Field For the Aspirator.**—A. H. Garnet, M.D., (Cincinnati Lancet and Clinic, January 6th, 1883), says, after having tried in vain to relieve a patient suffering from retention of urine, we were about at our wits' end when the aspirator was brought out. (Miller, with stomach pump combined.) The needle was detached and the rubber attached to a catheter, previously introduced into the bladder. The aspirator was then worked upon the principle for which it was devised, and the powerful suction not only dislodged the clots, but drew them through the instrument, and they were discharged along with the urine through the escape tube of the aspirator, to the relief of both doctor and the patient.—*Medical Index.*

**Digitalis to Prevent Excessive Desire for Copul tion.**—In the case of a man who had persistent desire to have intercourse with his wife, even during the day, Dr. Folsom reports in the Medical World the successful action of digitalis as a complete depressor of venereal appetite. He prescribed it in 10-drop doses, but the efficient point was attained only after a teaspoonful at a dose was reached, which abolished the power to cause erections, though it did not prevent the amative desire. In another case a young man wanted something that would enable him to hold his passion in check. The digitalis was prescribed for him also, commencing with ten drops, gradually increasing the dose until the desired effect was obtained, which required teaspoonful doses, as in the previous case. Dr. Folsom prescribes it in insanity resulting from self abuse.—*Buffalo Med. and Surg. Journal.*

**Treatment of Malaria.**—Dr. Wagginer, in Medical and Surgical Reporter, writes: "In my student days I was taught that malaria disappeared with the coming of frost, but I practice in a country which it seems had not at that time been studied. We have malarial diseases the year round. A freezing temperature lasting for days and weeks does not stop it. We have *typical nothing* except malarial diseases; all others are complicated, malaria being a conspicuous element. Hence, cases of chronic malarial poisoning are quite numerous. 'Tis quite common to have patients apply for treatment in this condition: Skin dry and sallow, bowels constipated, tongue furred, taste bad, urine scant and high colored, appetite capricious, daily exaltations of temperature without rigors, night-sweats, great nervousness and poor sleep, spleen enlarged to four or five times its normal size, more frequent in females, and especially so in those who have borne children; loss of strength and weight. In such cases, after checking periodicity and arousing secretion with mercurial purgative, cinchonidia is used almost to the exclusion of quinine here. I place patient upon one of the following:

℞ Tinct. iodinii comp.........................f. ℥ j,

Liqr. potass. arsen.........................f. ʒ ij $\frac{2}{3}$,

Ergot, ex. fld.........................f. ʒ v $\frac{1}{3}$,

M. A teaspoonful (ʒ j) for adult, after each meal.

Or,

℞ Potass iodidi......................... ʒ ij $\frac{2}{3}$,

Liqr. potass. arsen.........................f. ʒ ij $\frac{2}{3}$,

Vin. ferri amari.........................f. ℥ iv.

M. A teaspoonful (ʒ) after each meal.

The first I prefer, though sometimes alternate. Have known this to be taken four months consecutively. Have also used it in other glandular enlargements and fibroids, and noted its good effects beyond question.

"I have had abundant opportunities of trying these combinations in cases of chronic malaria with enlarged spleen, and so can testify to their superiority over any of the many other combinations which I have tried. I don't think I have ever seen either of the formulæ in print; if so, the fact has escaped my mind."

**Therapeutical Remarks on Hamamelis Virginica.**—A writer in Medical and Surgical Reporter says: "It has been with me, as no doubt with many others, a rule of action, that when the remedy of main reliance in the treatment of a disease proves, on trial after trial, unsatisfactory, not to rest until a better one is found—one that will fulfill all reasonable objects. It was in the search for such a remedy, as a local application for hemorrhoids, that I was led, some twelve years ago, to try the hamamelis. Palliatives in this disorder are frequently called for by those who firmly refuse all surgical interferences. After having tried almost every remedy mentioned by respectable authority for the above purpose, with very unsatisfactory results, I was induced to give the extract of witch-hazel a trial.

"The reports, almost invariably, were so favorable that the desideratum seemed to be at last attained. Its application not only lessened, but often wholly checked, the bleeding, besides assuaging the suffering to a notable degree. Of course, no benefit is looked for where the vessels become strangulated and inflamed; but when they can be readily returned, well anointed with the hamamelis, benefit is quickly perceptible. The pain and soreness are soon mitigated, and a striking diminution of their size is ere long apparent. Neither this application nor any other is of much benefit unless aggravating causes are avoided, such as very dry and long-retained fæces, diarrhœa, and obstructed or turgid states of the portal circulation. With these sources of aggravation removed, the fluid extract of hamamelis, with an equal portion of glycerine, and a little starch or other excipient, for convenience of application, well smeared over the piles, and these returned, will do all, and more than any other application. A lady who had been a great sufferer from a large cluster of bleeding piles for more than twenty years—now a little better, then worse—and who had tried every remedy told her, said that no application had equaled in relief the one I gave her. Of course, it only afforded, in such a severe and chronic case, decided mitigation, but it led her trustfully to submit to my judgment and get rid of the trouble permanently by the hypodermic use of a solution of persulphate of iron, which she did."

**The New Local Anæsthetic.**—Dr. C. R. Agnew, at a recent meeting of the New York Academy of Medicine, gave it as his opinion that since the discovery of anæsthesia by ether and chloroform, nothing had been given to surgery of more practical interest than the hydrochlorate of cocaine.

He described an operation for the removal of cataract by von

Graefe's method, performed upon an aged female at the Manhattan Hospital. A two-per-cent. solution was used, and three instillations of one drop each were made at intervals of five minutes. In nine minutes after the last instillation the operation was complete, and the bandage placed over the eye. All the inconveniences and dangers of a general anæsthetic were avoided. The drug produces mydriasis as a secondary effect, and the anæsthesia is of short duration.

Two of the fellows showed their faith in Dr. Agnew's word by submitting themselves to the *experimentum crucis.* They got each a drop in the eye, and had their conjunctivæ pinched with the forceps, without showing any sign of pain.

Dr. Burchard bore testimony to the painless opening of a felon after the affected finger had been held in the cocaine solution for some time.

It is not improbable that the new local anæsthetic will successfully substitute the general anæsthetic, in all cases of minor surgery at least.—*Louisville Med. News.*

**Fever.**—A writer in the American Medical Journal says: Having a little leisure time, I propose, if it meets your approval, to write a few lines for your journal. At this time of the year in the Southwest many of the cases met with by the country practitioner are of the so-called bilious remittent or typho-malarial fever. I have practiced in Texas, Arkansas, and Missouri since 1865, and treated hundreds of such cases during that time.

My experience has been, in this class of cases, to first produce an action of the liver, or carry off the surplus supply of bile, which may be done with several different remedies, according to the judgment of the practitioner. I generally give a good cathartic, and in the adult, say, five grains of sulphate of quinia, combined with two or three grains of powdered cloves, and add from one-sixth to one-eighth grain sulph. morphia; if the patient does not bear the quinia well, or chinchonism is easily produced, also the following is given: Nit. potas., pulvis opii et ipecac, co-equal quantities, say from three to five grains each. I commence with the quinia immediately, and alternate with the other every hour. In the morning, about daylight, commence with the quinia an hour after one of the comp. ipecac and opium powders, and so on through the day. I do not stop the quinia when the fever rises, but continue it, fever or no fever. In this manner, after one day's treatment like this, the patient will find, though somewhat weak, his fever is all gone. I think, from my experience of over twenty-three years' practice, you will find no case present any symptoms of continuance after twenty-four hours' treatment in this manner. As a matter of course, if any complications are present in the case, they will have to be attended to as the judgment of the physician may dictate. One reason why this mode of procedure is successful is, that the kidneys and skin are set to work and assisted to eliminate the fever-poison rapidly.

Do not be afraid to pile in the quinine rapidly because the fever rises, but continue just the same as though there were no fever. I

believe quinine acts better during the febrile condition than when it is off. Regulate your dose of the quinine to suit cases ; in many five grains will be too large ; if so, reduce it.

If you want to keep a patient on hand, so as to make a large bill, don't use the above, for it will not pay. Try this plan if you want to get your patients up rapidly, and you will be surprised, as well as find a pleased patient, on your return. As a matter of course, after the exacerbations are broken up, your judgment will dictate the necessary tonics, stimulants, etc., which follow to put the case on foot again.

**Typhoid Fever.**—Dr. S. K. Jackson, of Norfolk, Va., in a paper read before the American Medical Association, lays down the following law for the destruction of germs in disease, which, if true, is of very great importance to the profession :

" No organism can live in its own excreta, in the products of its own vital processes. When carbonic acid gas is the excretory product, carbonic acid will destroy the life that produced it. If alcohol be the result of the life process, what better agent have we for arresting that process than alcohol itself ?

" If sulphureted dydrogen be the exhaled secretion, all acknowledge the efficacy of sulphur and its compounds to arrest the decomposition giving rise to it. So also if ammonia be the result of the vital process of an organism, then by this law we have the right to infer ammonia to be the proper and efficient germicide."

Typhoid fever he claims to be the product of a microzone which is a consumer of nitrogen, as is shown by the ammoniacal exhalations emitted by a typhoid fever patient from the breath, skin, and urine, these exhalations being due to the decomposition of nitrogenous constituents caused by this micro-organism. In accordance with his law formulated above, he treats the case by the free administration of ammonia, even to the saturation of the system. In the high febrile condition of the early stages of the disease he gives the nitrate of ammonia in 10 or 12-grain doses, claiming that it will reduce the temperatnre to 102° Fahr., and keep it there during the whole course of the disease. As the disease progresses, especially if diarrhœa supervene, he substitutes the acetate for the nitrate, giving at the same time acetate of lead and opium. Should nervous symptoms show themselves, tongue become coated, teeth dry and covered with sordes, he resorts to carbonate of ammonia in combination with potassa chlorate. Should coma occur, he uses "hydrochlorate of ammonia, which" he " has never known to fail in bringing a case out of that condition." He says : " I have known them to be brought out of it when apparently in articulo mortis, when all treatment has been stopped and the case relinquished."

With this treatment he claims a success which is certainly phenomenal. In one epidemic, out of two hundred and twenty-two cases treated in this manner, not one died.

In conclusion, he says : " My experience with this fever, when treated by the plan here pointed out, enables me to expect that if a case be recognized as one of enteric fever within three days of its inception, it may break at the end of the first septenary. If it be not recognized as such until the fourth or fifth, it can not yield before, but may at the end of the second septenary. (Fourteenth day.) If the treatment has been commenced before the beginning of the second septenary, the fever cannot break before the end of the third (twenty-first day) ; that it will yield on that day, if it has not yielded before, is almost an absolute certainty."—*The Medical Index*.

**A New Hair Dye.**—The disadvantages attending the use of hair dyes containing lead, and the positive danger attending their use, have induced M. Maquet to search for a liquid which may be used for dyeing the hair and yet be inocuous. He describes, in the Moniteur Scientifique, a dye which is said to have progressive action, to produce all shades up to a deep chestnut color, and yet to be free from all deleterious action. The base of the dye is bismuth. The following is the formula : Bismuth is dissolved in the smallest possible quantity of nitric acid—nearly three parts—and to this liquor a solution in water of tartaric acid, equal in weight to one-fourth of the bismuth used, is added, and then a large quantity of water, so as to insure thorough precipitation of the bismuth. The precipitate is filtered off, and washed with water until the washings have lost all acidity. The precipitate is dissolved in a solution of ammonia ; and for this rather more than a fluid ounce of ammonia will be required for each ounce of bismuth used. Hyposulphite of soda—three-fourths of the weight of the bismuth employed—is then added, and when the salt is dissolved the mixture is filtered, and preserved in well-closed bottles. The dye should contain about one-twentieth of its weight of bismuth. Such a mixture is said to form an admirable dye, which loses ammonia on exposure to air, and deposits sulphite of bismuth.—*British Med. Journal.*

**Extirpation of Small Round Tumors of the Skin with a Quickly-rotating Punch.**—Instead of using an ordinary knife for the removal of little tumors of the skin, Dr. F. Busch (" Berl. klin. Wochenschr," xxi, 1884) advocates the use of a steel punch which is made to rotate rapidly either by attaching it to the dentist's drilling engine, or, more conveniently, by fastening it to Heurteloup's artificial leech. For different sized tumors different sized punches must be used ; but, as this method is best adapted to small tumors of one centermeter diameter or less, the size of the punch need never be large. The instrument having first been adjusted according to the depth to which it is desired to cut, the punch is placed over the tumor, a spring is liberated, and in an instant the excision is made. A clean circular wound is left, which should be dressed in the usual manner for open wounds, and be allowed to heal from the bottom. When operating upon loose tissue, such as the eyelid, the requisite resistance is obtained by putting the part on the stretch—in the case of the eyelid, by inserting a piece of wood or horn under it. The lip should be supported by the finger. The advantages stated for this method are the simplicity of the operation and the avoidance of anæsthetics.—*N. Y. Med. Jour.*

